# Identification and Development of a Novel 4-Gene Immune-Related Signature to Predict Osteosarcoma Prognosis

**DOI:** 10.3389/fmolb.2020.608368

**Published:** 2020-12-23

**Authors:** Mingde Cao, Junhui Zhang, Hualiang Xu, Zhujian Lin, Hong Chang, Yuchen Wang, Xusheng Huang, Xiang Chen, Hua Wang, Yancheng Song

**Affiliations:** ^1^Department of Orthopedics, The First Affiliated Hospital of Guangdong Pharmaceutical University, Guangzhou, China; ^2^Department of Orthopaedics and Traumatology, Faculty of Medicine, The Chinese University of Hong Kong, Shatin, Hong Kong

**Keywords:** gene signature, prognosis, osteosarcoma, immunotherapy, bioinformatics analysis

## Abstract

Osteosarcoma (OS) is a malignant disease that develops rapidly and is associated with poor prognosis. Immunotherapy may provide new insights into clinical treatment strategies for OS. The purpose of this study was to identify immune-related genes that could predict OS prognosis. The gene expression profiles and clinical data of 84 OS patients were obtained from the Therapeutically Applicable Research to Generate Effective Treatments (TARGET) database. According to non-negative matrix factorization, two molecular subtypes of immune-related genes, C1 and C2, were acquired, and 597 differentially expressed genes between C1 and C2 were identified. Univariate Cox analysis was performed to get 14 genes associated with survival, and 4 genes (*GJA5, APBB1IP, NPC2*, and *FKBP11*) obtained through least absolute shrinkage and selection operator (LASSO)-Cox regression were used to construct a 4-gene signature as a prognostic risk model. The results showed that high *FKBP11* expression was correlated with high risk (a risk factor), and that high *GJA5, APBB1IP*, or *NPC2* expression was associated with low risk (protective factors). The testing cohort and entire TARGET cohort were used for internal verification, and the independent GSE21257 cohort was used for external validation. The study suggested that the model we constructed was reliable and performed well in predicting OS risk. The functional enrichment of the signature was studied through gene set enrichment analysis, and it was found that the risk score was related to the immune pathway. In summary, our comprehensive study found that the 4-gene signature could be used to predict OS prognosis, and new biomarkers of great significance for understanding the therapeutic targets of OS were identified.

## Introduction

Osteosarcoma (OS) is the prevailing primary malignant tumor of the bone, and it is most likely to occur in children and adolescents, with 80–90% of OS tumors occurring in the metaphysis of the long tubular bones, distal femur, proximal tibia, and proximal humerus (Jackson et al., 2016). OS is highly malignant and develops rapidly, and it is likely to metastasize to the lung in the early stage (Pan et al., 2019). The average 5-year survival rate of those with local OS is about 80%, but the outcome for those with metastatic disease is much worse (Mirabello et al., 2009). Surgery, chemotherapy, and radiotherapy developments play an extremely important role in reducing the rate of lung metastasis and improving the long-term survival rate of patients with OS, making the 5-year survival rate reach 60–80% (Jawad et al., 2011). Targeted therapy and immunotherapy may provide new opportunities for comprehensive OS treatment. In recent years, the discovery of immune checkpoints has pushed cancer immunotherapy to a new level, with specific blocking of immunosuppression effects and enhanced anti-tumor immune responses. As an emerging treatment, immunotherapy has shown promising results for OS (Miwa et al., 2019). However, there are still many problems to be solved in immunotherapy for OS, especially in terms of predicting immunotherapy biomarkers and identifying new effective therapeutic targets.

Immunotherapies, particularly those based on immune checkpoint inhibitors, have shown promising activity in a variety of tumors, with clinically significant improvements in response rates, progression-free survival, and overall survival in lung cancer, head and neck cancer, and bladder cancer (Chen et al., 2017). In many malignancies, innate and adaptive immune cells play a role in the tumor microenvironment, communicating with antigen-presenting cells, such as natural killer cells, macrophages, and dendritic cells, as well as lymphocytes, thereby allowing for effective tumor control (Gajewski et al., 2013). Recently, immunotherapy has shown promise in a variety of cancers, but its application in OS remains unexplored. It has been suggested that OS may be sensitive to immunotherapies. The percentage of CD8+ infiltrating lymphocytes in OS is higher than in other sarcoma subtypes (van Erp et al., 2017), and the degree of infiltration is positively correlated with survival (Gomez-Brouchet et al., 2017). Some studies have shown that the use of immune checkpoints can be a promising treatment for OS. High PD-L1 levels have been observed in patients with OS, and PD-L1 levels have been associated with tumor-infiltrating lymphocyte levels (Shen et al., 2014). In addition, the median overall survival of patients with low PD-L1 levels was shown to be longer than that of patients with high PD-L1 levels. A recent study reported that PD-1 levels in peripheral blood CD4+ and CD8+ T cells in patients with OS were high and that PD-1 levels in CD4+ T cells in patients with metastasis were significantly higher than in patients without metastasis, and PD-1 and PD-L1 levels were found to be negatively correlated with prognosis (Zheng et al., 2015). OS has high levels of genomic instability, and some tumors express PD-L1, suggesting their potential sensitivity to inhibitors of the PD-1/PD-L1 axis (Hacohen et al., 2013; Kansara et al., 2014; Mouw et al., 2017). This all suggests that PD-1 is involved in the pathogenesis and progression of OS. Further, a prospective, randomized phase III trial showed that muramyl tripeptide phosphatidyl-ethanolamine, which is an investigational agent only available in clinical trials, could activate monocytes, and macrophages to improve tumor control. The addition of muramyl tripeptide to chemotherapy was shown to increase the 6-year overall survival rate of patients with OS from 70 to 78%, and EFS tended to improve (Meyers et al., 2008). In summary, several biological characteristics of OS suggest that regulation of the immune response may bring benefits, and a better understanding of OS may provide new insights for immunotherapy in terms of antibody targeting of cell surface proteins, tumor vaccines using dendritic cells, oncolytic viruses, adoptive cell therapy, and checkpoint inhibitors (Wedekind et al., 2018). It is hoped that immunotherapy will lead to a breakthrough in OS therapy.

To increase immunotherapy efficacy, it is especially pivotal to determine immune-associated prognostic biomarkers. OS is characterized by high heterogeneity of somatic copy number changes and structural rearrangement, suggesting that genes included in somatic copy number changes are the key drivers of cancer (Sayles et al., 2019). By focusing on genes that have an underlying role, we were able to reduce the complexity of the OS genomic pattern to identify the changes most likely to be relevant to clinical practice. This suggested the need for genomic information to perform targeted therapy. Here, we combined the expression data derived from multiple OS genes from the Therapeutically Applicable Research to Generate Effective Treatments (TARGET) and Gene Expression Omnibus (GEO) databases, and we used the non-negative matrix factorization (NMF) method to perform molecular subtyping based on immune-related genes. Further, we used a robust likelihood-based survival model to develop a 4-gene signature to predict OS prognosis. A prognostic risk score model was established, and internal and external verifications were performed. Our study may provide new biomarkers for predicting the therapeutic efficacy of immunotherapy and OS prognosis.

## Materials and Methods

### Data Sources and Preprocessing

A total of 84 OS samples with RNA sequencing (RNA-Seq) data and corresponding clinical follow-up information were downloaded from the TARGET database (https://ocg.cancer.gov/programs/target), and the GSE21257 cohort containing 53 OS samples with prognostic information was downloaded from the GEO database (www.ncbi.nlm.nih.gov/geo).

The patients collected from the TARGET database were used for the expression profiles sequenced by RNA-Seq, while the GSE21257 cohort was used for chip data (the platform was the Illumina Human-6 v2.0 Expression BeadChip). In the probe design process, some genes were not designed to be detected as probes. For the RNA-Seq data, different genomic backgrounds could be different, so the genes detected by the different data platforms could also be somewhat different.

In addition, 864 genes were collected from the immune-related literature (Nirmal et al., 2018). We preprocessed the TARGET dataset to filter for immune-related genes with low expression. The filter criterion was to remove genes with expression <1, which accounted for more than 50% of all samples. A total of 60 genes were removed from the TARGET data due to low expression. The details of those genes are given in Supplementary Table 1. The clinical features of the samples are provided in Table 1.

**Table 1:** Sample clinical features.

**Clinical features**	**TARGET-OS**	**GSE21257**
**Status**		
Censored	55	30
Dead	29	23
**Gender**		
Male	47	34
Female	37	19
**Metastatic**		
Yes	21	34
No	63	19
**Age**		
≤15	46	21
>15	38	32

### Identification of Molecular Subtypes Based on Immune-Related Genes

We extracted the expression profiles of 804 immune-related genes from the TARGET database, then univariate Cox analysis was performed using the coxph function to acquire prognosis-related genes. NMF was applied to cluster the OS samples, with the standard “brunet” and 50 iterations. We set the number of clusters, k, from 2 to 10, and we determined the average silhouette width of the co-membership matrix by using the NMF package in R, with the minimum member of each subclass set to 10. Kaplan–Meier (KM) survival curves were drawn to compare prognoses between the molecular subtypes. The limma package in R was used to identify the differentially expressed genes (DEGs) between the different molecular subtypes, and the ClusterProfiler package was used to perform Kyoto Encyclopedia of Genes and Genomes (KEGG) enrichment analysis of the DEGs.

### Construction of the Prognostic Risk Model

#### Random Grouping of Samples

The expression profiles of 597 DEGs between the molecular subtypes were retained. A total of 84 OS samples from the TARGET database were divided into a training cohort and a testing cohort. To avoid random allocation bias from affecting the stability of subsequent modeling, all samples were randomly grouped with replacement 100 times in advance. Group sampling was based on a training cohort-to-verification cohort ratio of 0.5:0.5.

#### Univariate Cox Analysis of the Training Cohort

Univariate Cox proportional hazards regression was performed for each immune-related gene in the training cohort, and the coxph function of the survival package in R was performed with *P* < 0.01 as the threshold for filtering.

#### LASSO-Cox Analysis

To reduce the number of genes in the risk model, we performed least absolute shrinkage and selection operator (LASSO) regression. LASSO is a penalized regression method that reduces overfitting by performing shrinkage and model selection simultaneously. A more refined model is obtained by constructing a penalty function, which can compress some coefficients and set others equal to 0. Therefore, it retains the advantage of subset contraction. Further, it is a biased estimate for processing data with multicollinearity, which can realize the selection of variables while estimating parameters and better solve the multicollinearity problem in regression analysis.

### Relationships Between Risk Scores and Pathways

To observe the relationships between the risk scores of the different samples and biological functions, we selected the gene expression profiles corresponding to these samples. The GSVA package in R was applied for single-sample gene set enrichment analysis (ssGSEA). We calculated the score of each sample for different functions, thus obtaining the ssGSEA score of each sample corresponding to each function. Further, we observed the correlations between these functions and risk scores.

## Results

### Flow Chart of Analysis

**Figure 1:**
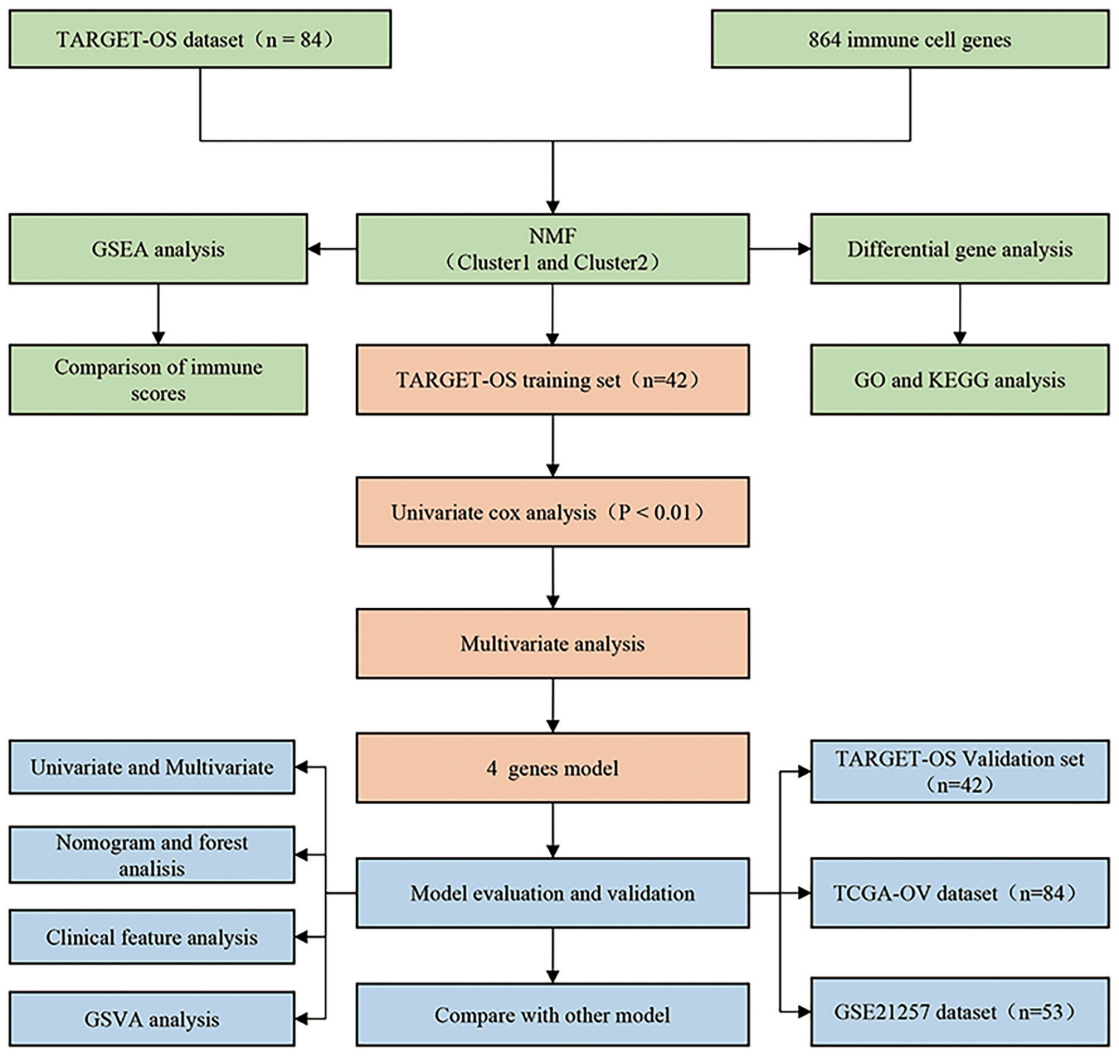
Osteosarcoma research protocol.

We designed a protocol for the analysis of OS subtypes and construction of the prognostic model. The analysis process was carried out in strict accordance with the protocol (Figure 1).

### Identification of Molecular Subtypes Based on Immune-Related Genes

**Figure 2:**
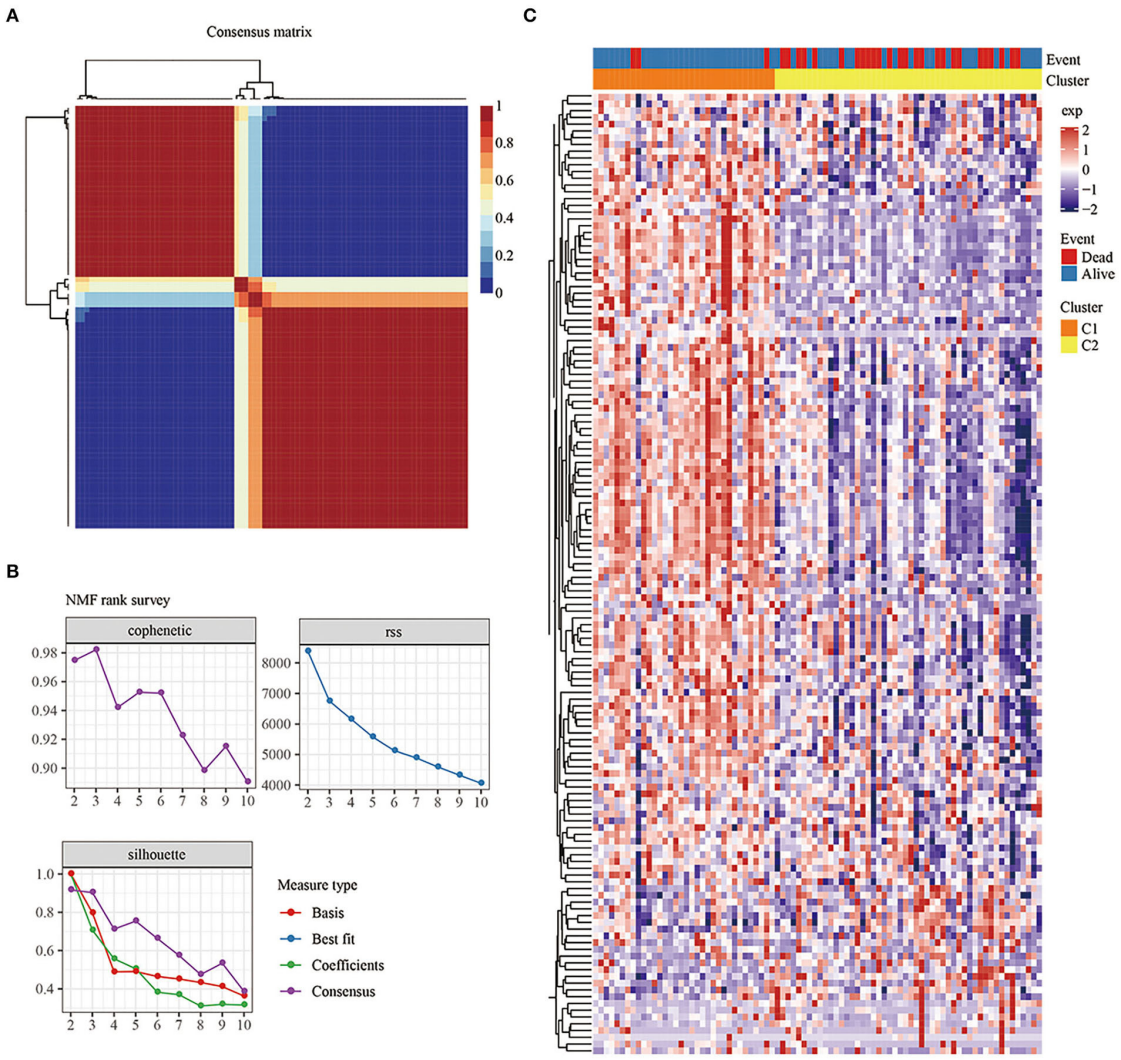
**(A)**: Consensus map of NMF clustering. **(B)** Cophenetic, rss, and dispersion distributions with ranks from 2 to 10. Cophenetic correlation is obtained based on the concordance matrix proposed by Brunet et al. and it is used to reflect the stability of the clusters obtained from NMF. The value of cophenetic correlation is between 0 and 1, and the larger the value, the more stable the clusters. The rss refers to the residual sum of squares, which is used to reflect the clustering performance of the model. The smaller the value, the better the clustering performance of the model. Theoretically, this value is the smallest when each sample is clustered into 1 class, but such results are not available in practice, so it needs to be used in combination with other indicators. **(C)** Heat map of the expression of the prognosis-related genes in the different subtypes.

Univariate Cox analysis was used to obtain 142 genes related to prognosis (*P* < 0.05; Supplementary Table 2). According to indicators such as cophenetic, dispersion, and silhouette in the NMF method, k=2 was chosen as the optimal number of clusters (Figures 2A,B). At the same time, cluster analysis was conducted on the expression profiles of these 142 genes (Figure 2C). Compared with the Cluster 2 (C2) subtype, most genes were highly expressed in the Cluster 1 (C1) subtype.

### Prognosis, Immune Score, and Biological Functional Analysis Between Subtypes

**Figure 3:**
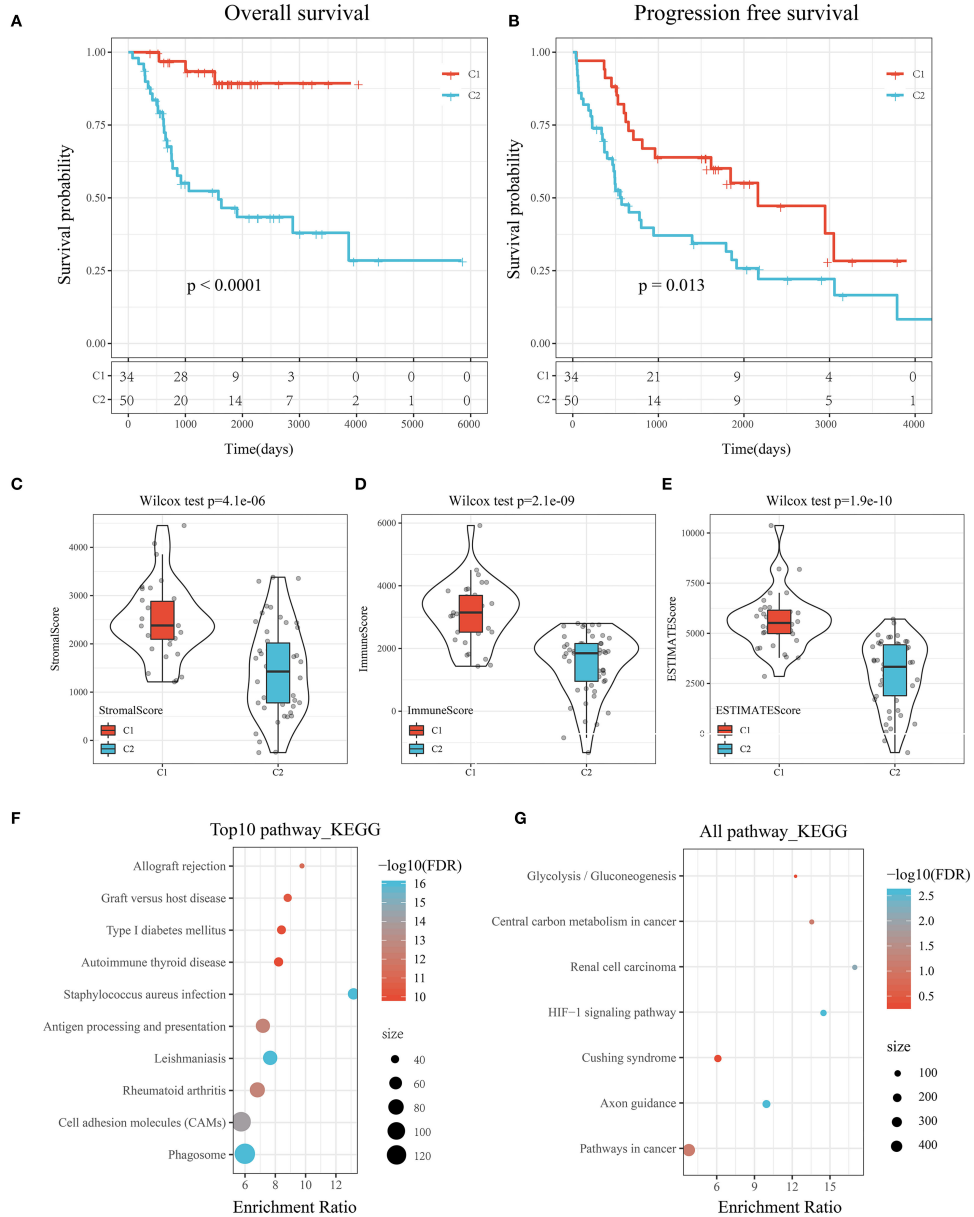
**(A)** KM curve of the overall survival of the subtypes of the TARGET tumor samples. **(B)** KM curve of the progression-free survival of the subtypes of the TARGET tumor samples. **(C)** Comparison of stromal scores between C1 and C2. **(D)** Comparison of immune scores between C1 and C2. **(E)** Comparison of ESTIMATE scores between C1 and C2. **(F)** KEGG pathway analysis of the upregulated DEGs between the molecular subtypes. **(G)** KEGG pathway analysis of the downregulated DEGs between the molecular subtypes.

KM survival curves were drawn to compare the survival of the two molecular subtypes. The results showed that C2 had worse prognosis than C1, for both overall survival and progression-free survival (*P* < 0.05; Figures 3A,B).

To determine the relationships of the immune and stromal scores between the two molecular subtypes, we estimated the immune and stromal scores of each sample by using the R software package and then made comparisons. The results showed that there were significant differences in stromal scores, immune scores, and ESTIMATE scores between C1 and C2, and the immune score of C1 was significantly higher than that of C2 (*P* < 1e-5; Figures 3C–E).

GSEA was performed to analyze the significantly enriched pathways in C1 and C2. More immune-related pathways were enriched in C1, including KEGG_B_CELL_RECEPTOR_SIGNALING_PATHWAY, KEGG_T_CELL_RECEPTOR_SIGNALING_PATHWAY, KEGG_TOLL_LIKE_RECEPTOR_SIGNALING_PATHWAY, and KEGG_NATURAL_KILLER_CELL_MEDIATED_CYTOTOXICITY, suggesting that C1 had a close relationship with immunity (Supplementary Figure 1).

Compared with C2, there were 597 DEGs, including 552 upregulated genes and 45 downregulated genes, in C1. The results suggested that the C1 and C2 subtypes were dominated by upregulated DEGs (Supplementary Table 3).

Further, KEGG pathway analysis of the DEGs between the subtypes of the TARGET data was performed by using ClusterProfiler (v3.14.0) in R. For the upregulated DEGs, a total of 62 pathways were annotated, among which 46 had a significant difference (false discovery rate <0.05). The top 10 results are shown in Figure 3F. Natural killer cell-mediated cytotoxicity, the toll-like receptor signaling pathway, the T cell receptor signaling pathway, the B cell receptor signaling pathway, and other immune-related pathways were significantly enriched (Supplementary Table 4).

Similarly, seven pathways were annotated among the downregulated DEGs shown in Figure 3G. The HIF-1 signaling pathway, pathways in cancer, and central carbon metabolism in cancer were enriched (Supplementary Table 5).

### Construction of The Prognostic Risk Model Based on Immune-Related Genes

#### Random Grouping of Samples and Univariate Cox Analysis

Group sampling was based on a training cohort-to-verification cohort ratio of 0.5:0.5. There were 42 training cohort samples and 42 testing cohort samples (Supplementary Table 6). Univariate Cox proportional hazards regression was performed for each immune-related gene. The results showed that there were 14 prognostic genes in the training cohort (Supplementary Table 7).

#### Construction of the Gene Signature by LASSO-Cox Analysis

**Figure 4:**
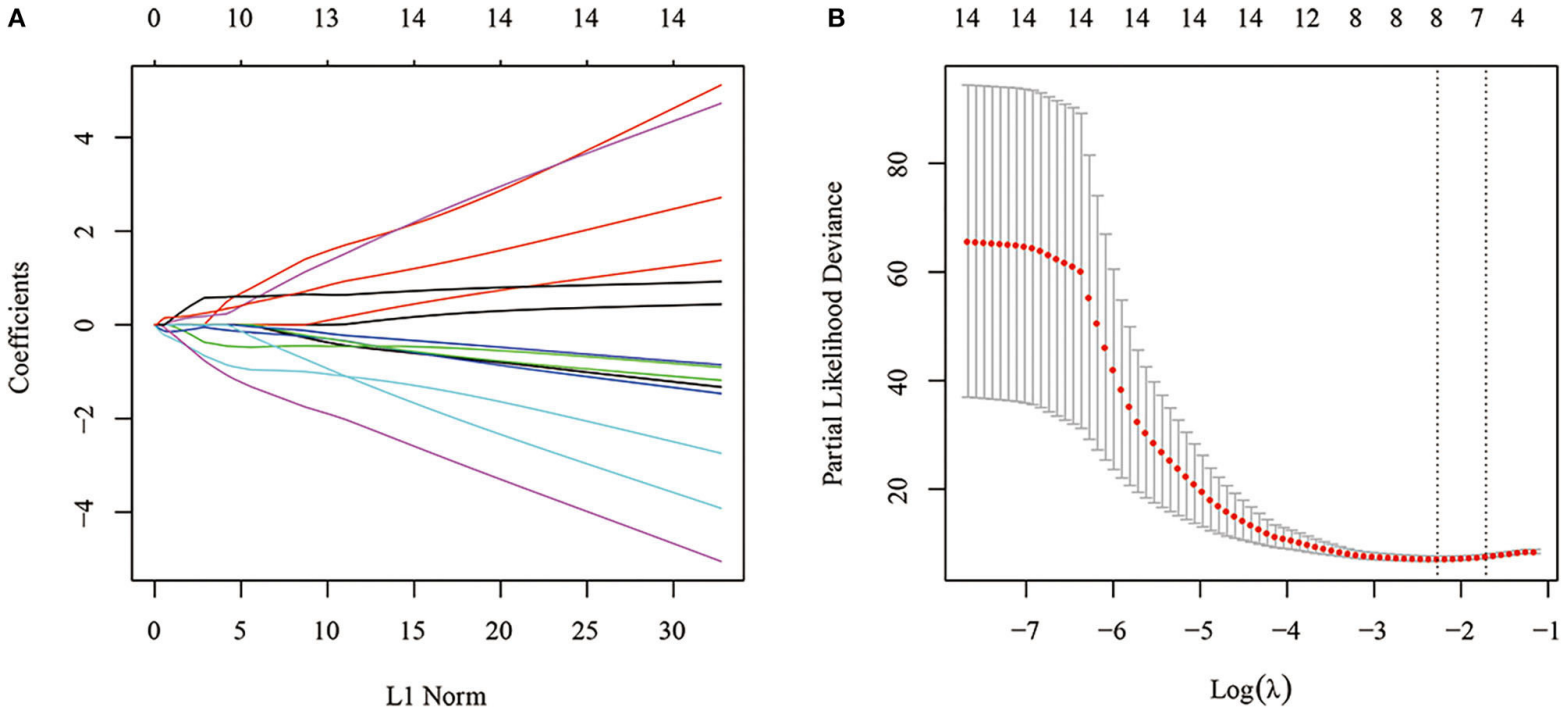
**(A)** Change trajectory of each independent variable. The horizontal axis represents the log value of the independent variable lambda, and the vertical axis represents the coefficient of the independent variable. **(B)** Confidence intervals under each lambda.

Fourteen was considered a large number of prognostic genes and inappropriate for the clinical context, so LASSO-Cox regression analysis was performed to further narrow the range of immune-related genes while maintaining a high accuracy rate. First, the change trajectory of each independent variable was analyzed (Figure 4A). The results showed that the number of independent variable coefficients approaching 0 increased with the gradual increase of lambda. A 10-fold cross-validation was used to construct the model. The confidence interval of each lambda is shown in Figure 4B. A total of 8 genes were selected as the target genes for subsequent analysis when the model reached the optimal value, with lambda equal to 0.1030521.

To further reduce the number of genes, the Akaike information criterion (AIC) was used for stepwise regression. AIC takes into account a model's goodness of fit and its simplicity in terms of the number of parameters needed to achieve this fit. The stepAIC function of the MASS package in R started with the most complex model and successively deleted variables in turn to reduce the AIC. The smaller the value, the better the model. It gave the model sufficient fit with fewer parameters. Finally, a total of 4 genes, *GJA5, APBB1IP, NPC2*, and *FKBP11*, was obtained.

**Figure 5:**
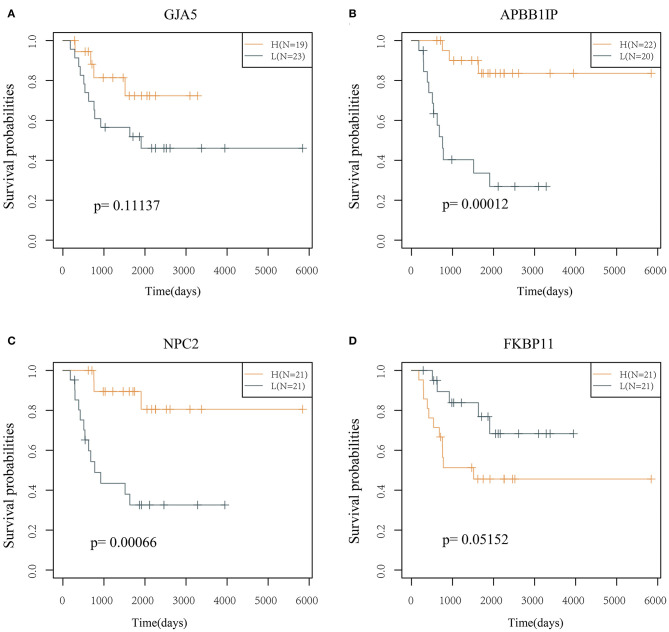
KM curve of the 4 genes (in the TARGET training cohort). **(A)** The survival curve of GJA5 gene. **(B)** The survival curve of APBB1IP gene. **(C)** The survival curve of NPC2 gene. **(D)** The survival curve of FKBP11 gene. The abscissa represents survival time, and the ordinate represents survival probability.

The prognostic KM curves of the 4 genes are shown in Figures 5A–D. *APBB1IP* and *NPC2* could divide the TARGET training cohort samples into high- and low-risk groups with prognostic significance (*P* < 0.05). *FKBP11* had marginal prognostic significance (*P* = 0.05152), and *GJA5* had no prognostic significance (*P* > 0.05).

#### Evaluation of the Risk Model

**Figure 6:**
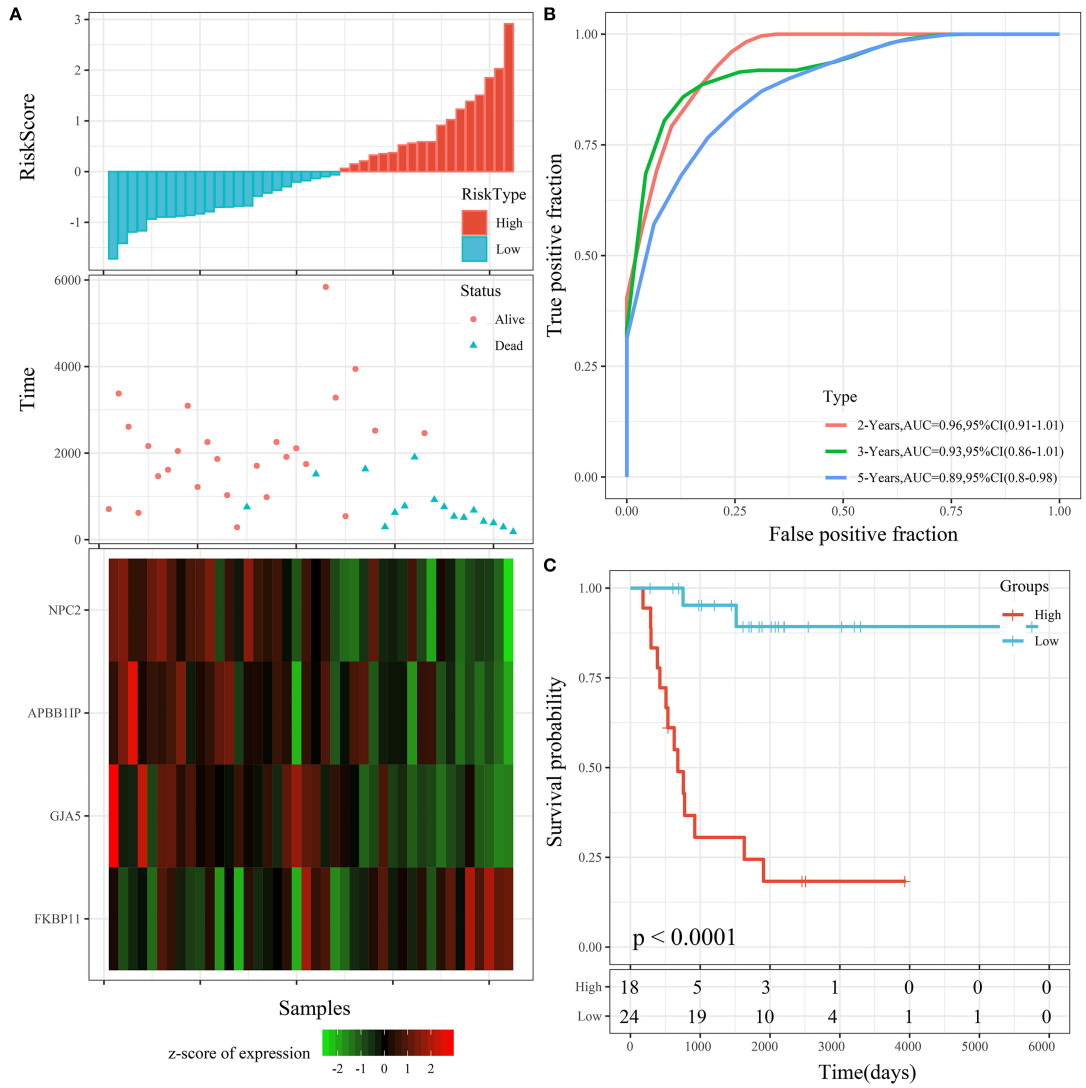
**(A)** Risk score, survival time, survival status, and 4-gene expression in the TARGET training cohort. **(B)** ROC curve and AUC of the 4-gene signature. **(C)** KM survival curve distribution of the 4-gene signature in the TARGET training cohort.

The 4 genes were used to construct the risk model, as follows: RiskScore=-0.873^*^GJA5-1.016^*^APBB1IP-1.192^*^NPC2+0.915^*^FKBP11. The risk score of each sample according to expression was calculated and shown as the risk score distribution (Figure 6A). The overall survival of the samples with high risk scores was significantly lower than that of the samples with low risk scores, suggesting that samples with high risk scores had worse prognoses. It was determined that the high expression of *FKBP11* was correlated with high risk (a risk factor), while the high expression of *GJA5, APBB1IP*, or *NPC2* was associated with low risk (protective factors). Further, receiver operating characteristic (ROC) analysis was conducted by using the timeROC package in R for the prognostic classification of risk scores. We analyzed the classification efficiency of prognosis prediction at 2, 3, and 5 years. The average area under the curve (AUC) reached 0.93 (Figure 6B). Finally, the *Z*-score method was applied to the preprocessing of risk scores. Samples with a risk score >0 were put into the high-risk group, while those with a risk score <0 were put into the low-risk group. A KM curve between the 2 risk groups was plotted (Figure 6C). There was a very significant difference (*P* < 0.0001), with 18 samples classified as high risk and 24 as low risk.

### Robustness Verification of The Risk Model

#### Risk Model Validation in the Testing Cohort and Entire TARGET Cohort

To determine the robustness of the model, we used the same model and the same coefficient in the training cohort for the testing cohort and entire TARGET cohort. We calculated the risk score of each sample according to expression and then plotted the risk score distribution.

**Figure 7:**
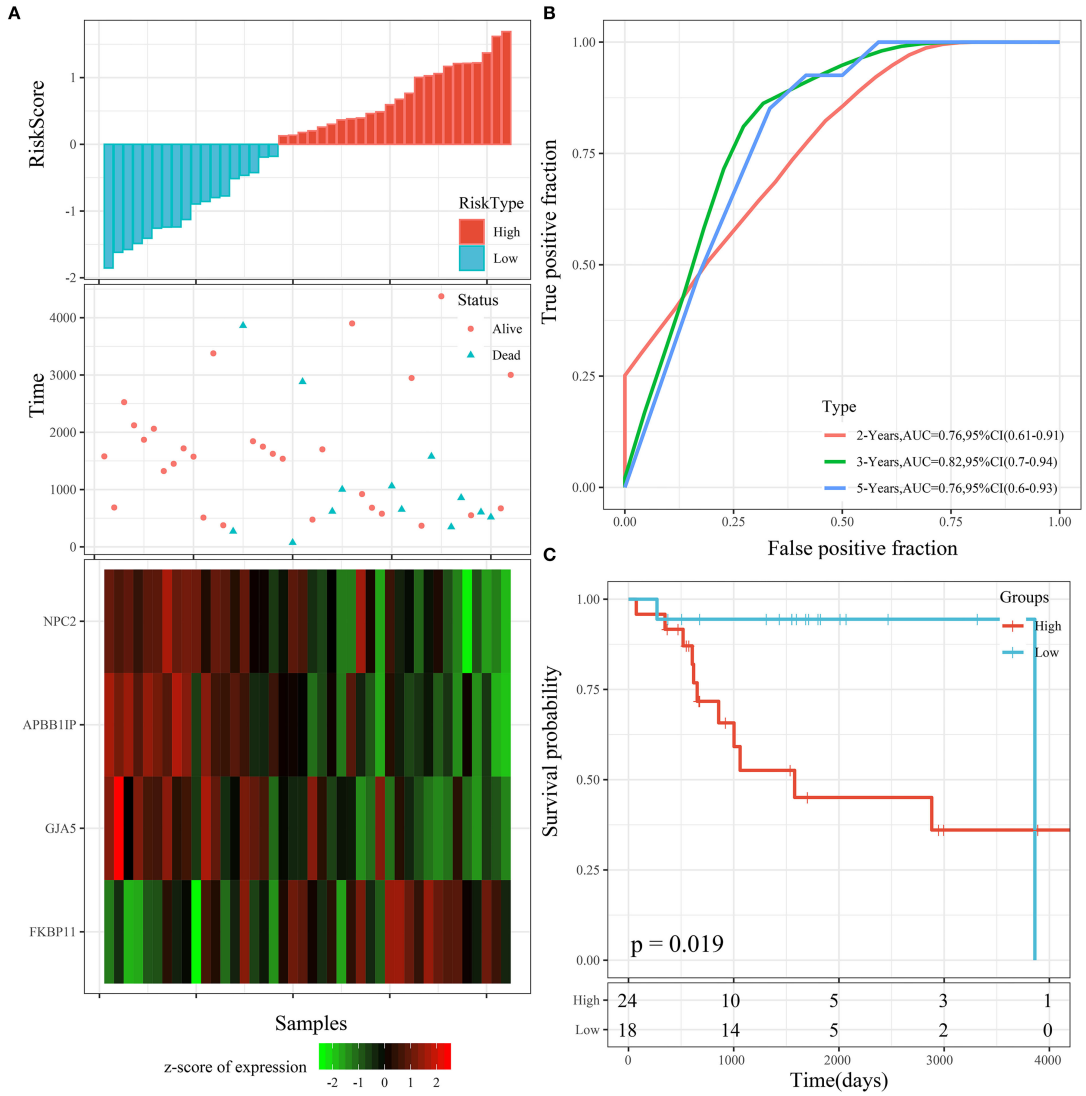
**(A)** Risk score, survival time, survival status, and 4-gene expression in the TARGET testing cohort. **(B)** ROC curve and AUC of the 4-gene signature. **(C)** KM survival curve distribution of the 4-gene signature in the TARGET testing cohort.

The risk score distribution of the testing cohort is shown in Figure 7A. The overall survival of the samples with high risk scores was significantly lower than that of the samples with low risk scores, suggesting that the samples with high risk scores had worse prognoses. Regarding changes in the expression of the 4 signature genes with increased risk scores, it was determined that the high expression of *FKBP11* was correlated with high risk (a risk factor). The high expression of *GJA5, APBB1IP*, or *NPC2* was associated with low risk (protective factors). This was consistent with the TARGET training cohort. The average AUC reached 0.78 (Figure 7B). The KM curve showed a very significant difference (*P* = 0.019), with 24 samples classified as high risk and 18 as low risk (Figure 7C).

**Figure 8:**
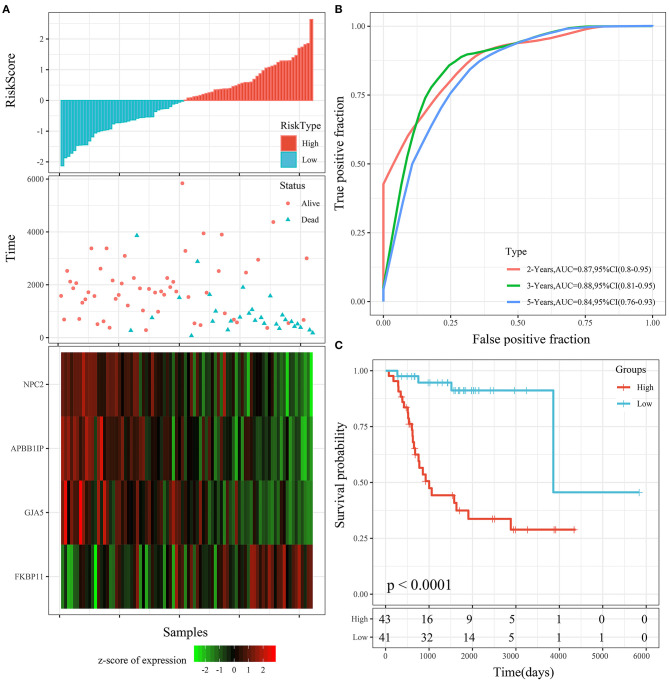
**(A)** Risk score, survival time, survival status, and 4-gene expression in the entire TARGET cohort. **(B)** ROC curve and AUC of the 4-gene signature. **(C)** KM survival curve distribution of the 4-gene signature in the entire TARGET cohort.

Th risk score distribution of the entire TARGET cohort is shown in Figure 8A. It was consistent with the performance of the TARGET training cohort. The average AUC reached 0.88. Prognoses in the high-risk group were significantly worse than those in the low-risk group (*P* < 0.001; Figure 8B). The KM curve showed a very significant difference (*P* < 0.0001), with 43 samples classified as high risk and 41 as low risk (Figure 8C).

#### Risk Model Validation in the External Cohort

**Figure 9:**
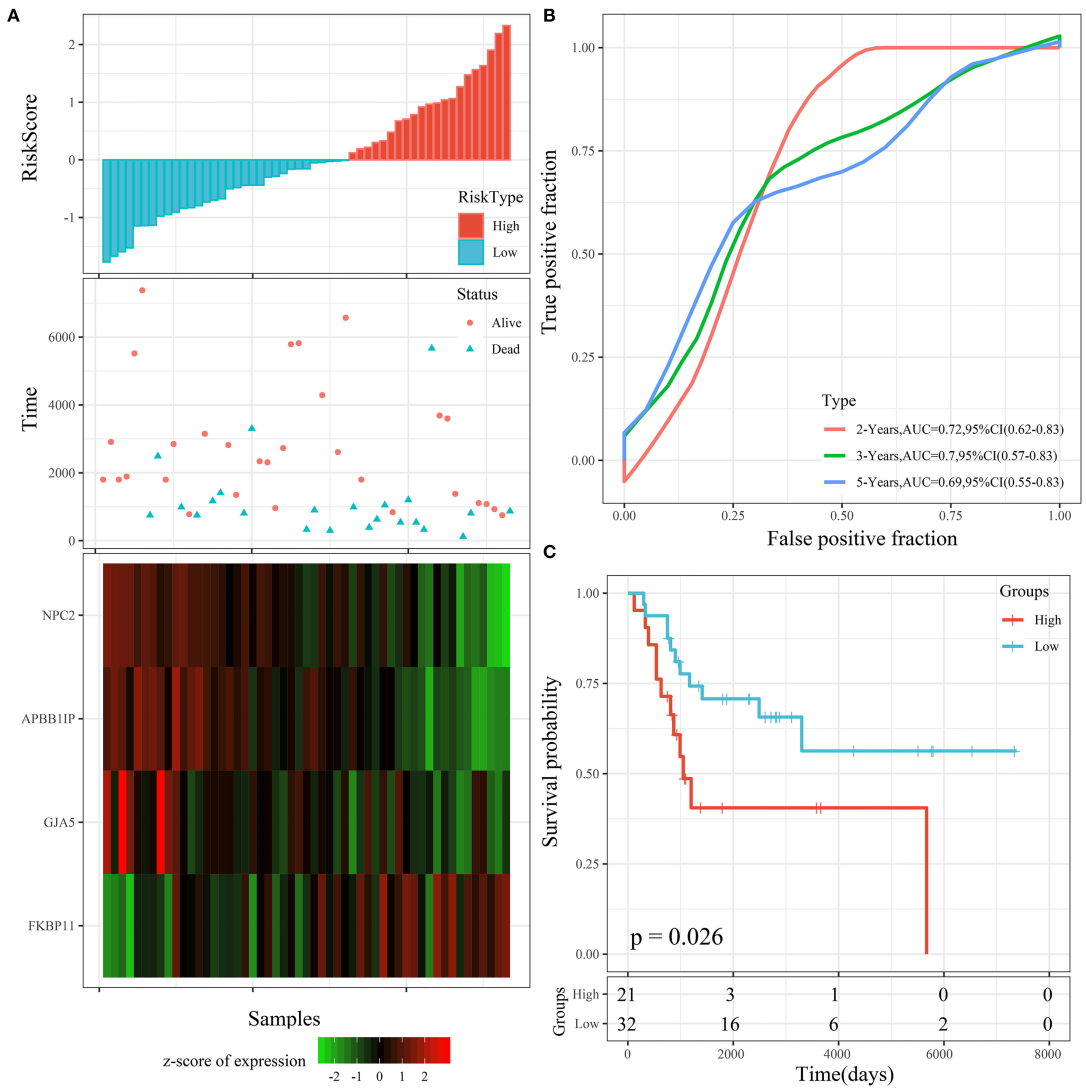
**(A)** Risk score, survival time, survival status, and 4-gene expression in the GSE21257 testing cohort. **(B)** ROC curve and AUC of the 4-gene signature. **(C)** KM survival curve distribution of the 4-gene signature in the GSE21257 testing cohort.

To determine the robustness of the model, we used the same model and the same coefficient in the training cohort for the external testing cohort. The risk score distribution of the GSE21257 testing cohort is shown in Figure 9A. The results were consistent with those of the TARGET training cohort. The average AUC reached 0.7 (Figure 9B). Prognoses in the high-risk group were worse than those in the low-risk group. The KM curve showed a very significant difference (*P* = 0.026), with 27 samples classified as high risk and 24 as low risk (Figure 9C).

### Prognostic Analysis of the Risk Score Model and Clinical Features

**Figure 10:**
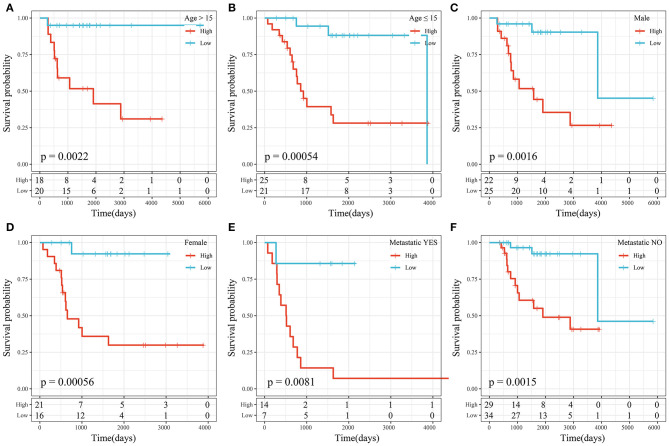
**(A)** KM curve of high- and low-risk samples with age >15 years. **(B)** KM curve of high- and low-risk samples with age ≤ 15 years. **(C)** KM curve of high- and low-risk samples of male sex. **(D)** KM curve of high- and low-risk samples of female sex. **(E)** KM curve of high- and low-risk samples with metastasis. **(F)** KM curve of high- and low-risk samples without metastasis.

We performed subgroup survival analysis of the clinical variables based on risk scores and found that the 4-gene signature divided the samples into the high- and low-risk groups appropriately in terms of age, sex, and metastasis (*P* < 0.01; Figure 10). The results suggested that the risk score model had good predictive ability in terms of different clinical features.

### Relationships Between Risk Scores and Pathways

**Figure 11:**
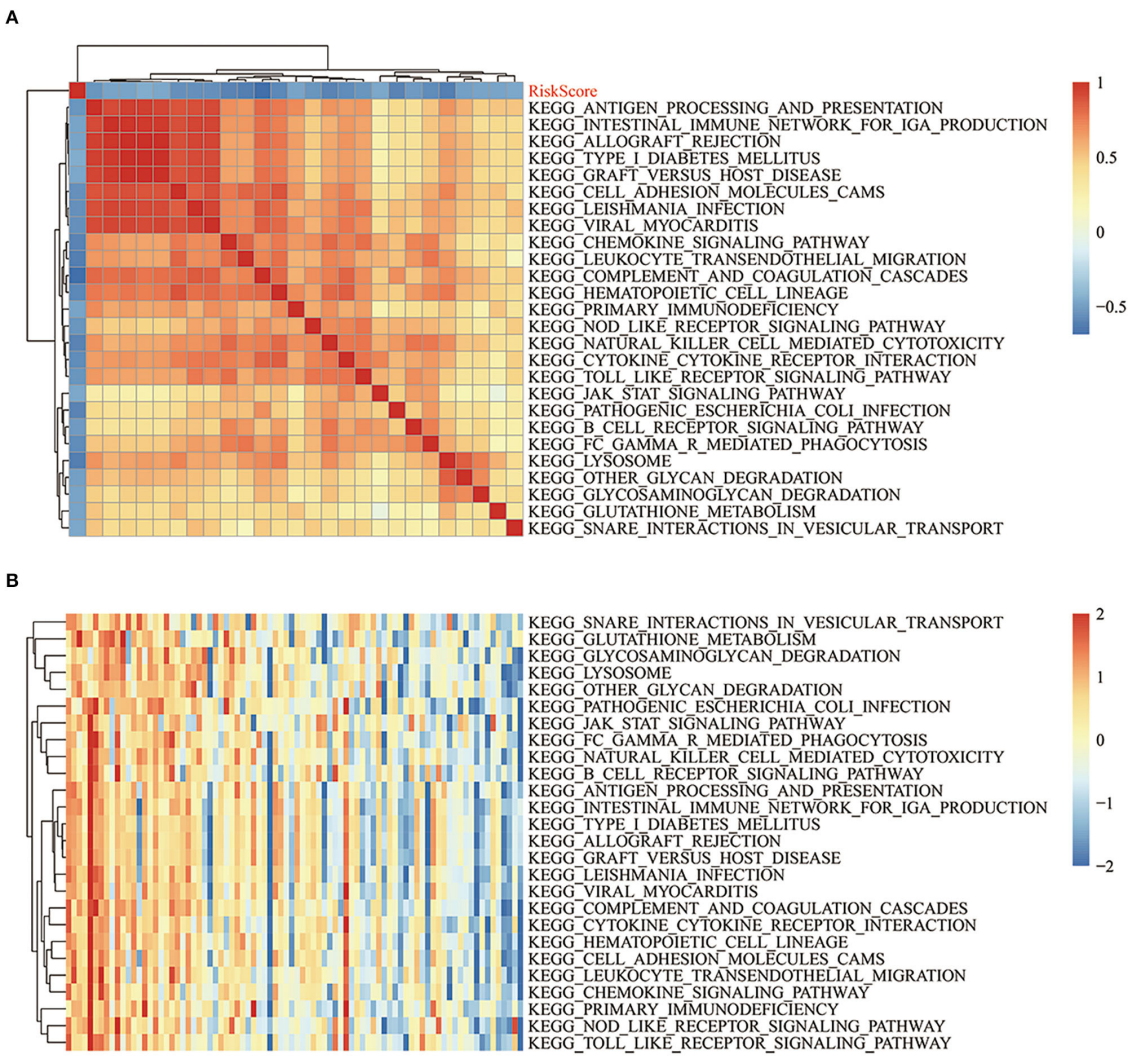
**(A)** Clustering of KEGG pathways with correlation coefficients >0.35 and risk scores. **(B)** ssGSEA score changes of each sample in regards to the KEGG pathways with correlation coefficients >0.35 and increased risk scores. The horizontal axis represents the sample, and the risk score increases successively from left to right.

To observe the relationships between the risk scores of the different samples and biological functions, ssGSEA was performed by using the GSVA package in R. The functions with correlation coefficients >0.35 were selected (Figure 11A). A total of 26 pathways were negatively correlated with risk score. The KEGG pathways used for cluster analysis based on enrichment scores are shown in Figure 11B. Some of the 26 pathways negatively correlated with increased risk score, including KEGG_JAK_STAT_SIGNALING_PATHWAY, KEGG_NATURAL_KILLER_CELL_MEDIATED_CYTOTOXICITY, KEGG_TOLL_LIKE_RECEPTOR_SIGNALING_PATHWAY, and KEGG_B_CELL_RECEPTOR_SIGNALING_PATHWAY.

### Univariate and Multivariate Cox Analyses of the 4-Gene Signature

Univariate and multivariate Cox regression analyses were used to determine the independence of the 4-gene signature model in clinical applications (Table 2). Univariate Cox regression analysis results showed that risk score was significantly correlated with prognosis; at the same time, multivariate Cox analysis results showed that it was an independent prognostic risk factor (hazard ratio = 2.9103, 95% confidence interval = 1.8364–4.6120, *P* = 5.4e-06). Forest maps can simply and intuitively display the statistical summary results of different factors. The multivariate Cox results in a forest diagram are shown in Supplementary Figure 2. The results suggested that the 4-gene signature model had good predictive performance in clinical applications.

**Table 2:** Univariable analysis and multivariable analysis of the entire TARGET.

**Variables**	**Univariable analysis**				**Multivariable analysis**			
	**HR**	**95% CI of HR**		***P***	**HR**	**95% CI of HR**		***P***
		**Lower**	**Upper**			**Lower**	**Upper**	
Age	0.9901	0.9118	1.0750	0.8130	1.0191	0.9298	1.1170	0.6858
Gender	0.6870	0.3304	1.4290	0.3150	0.6521	0.3005	1.4150	0.2793
Metastatic	4.7400	2.2710	9.8950	3.4E-05	3.1864	1.4696	6.9090	0.0033
RiskScore	3.4030	2.1600	5.3610	1.3E-07	2.9103	1.8364	4.6120	5.4E-06

### Comparison of the Risk Score Model With Other Models

**Figure 12:**
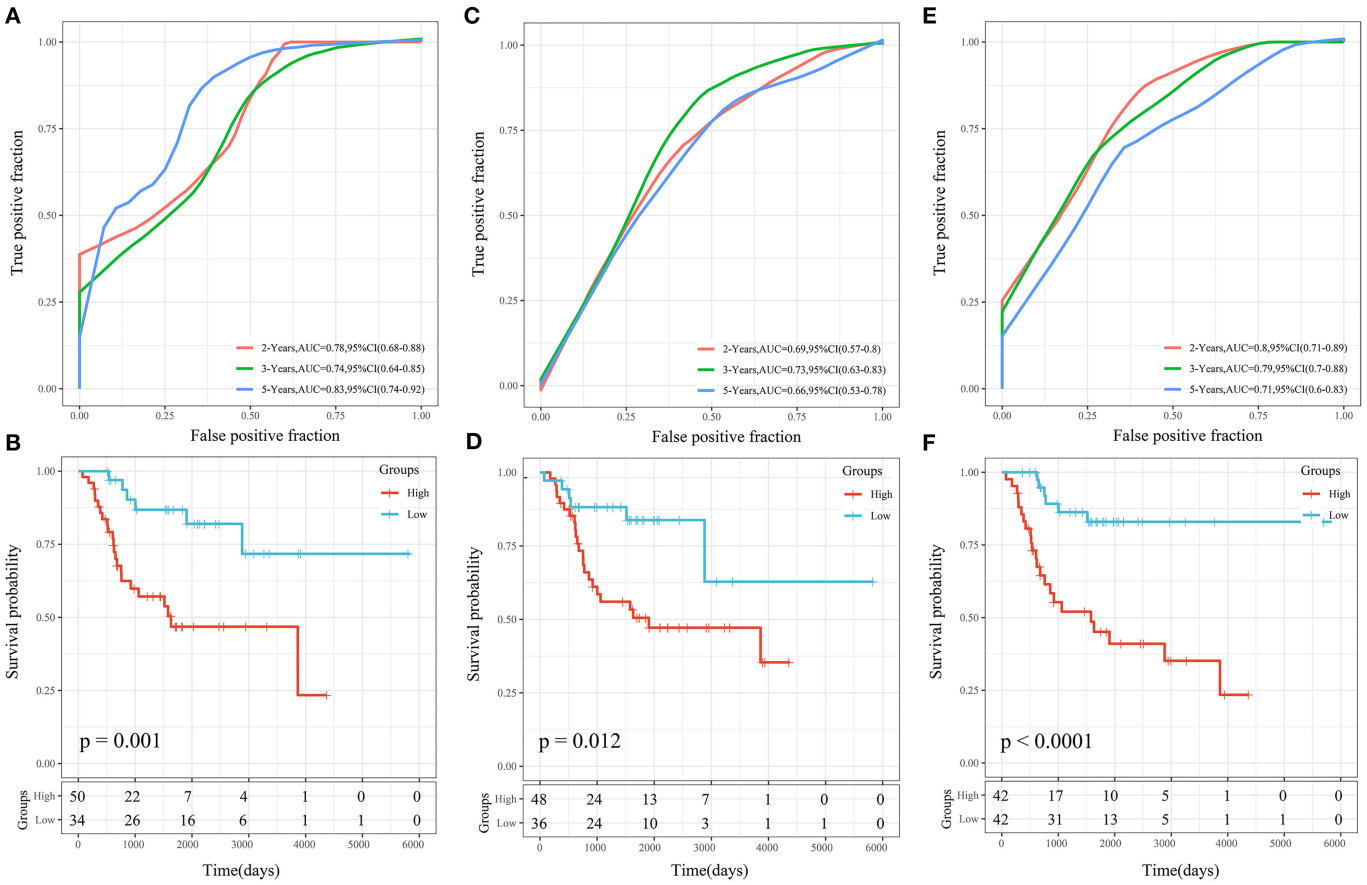
**(A,B)** ROC and KM curves of the overall survival of the high- and low-risk samples using the 19-gene signature risk model (Goh). **(C,D)** ROC and KM curves of the overall survival of the high- and low-risk samples using the 8-gene signature risk model (Zhang). **(E,F)** ROC and KM curves of the overall survival of the high- and low-risk samples using the 3-gene signature risk model(Shi).

Three OS prognosis-related risk models were identified by reviewing the literature, including a 19-gene signature (Goh et al., 2019), an 8-gene signature (Zhang et al., 2019), and a 3-gene signature (Shi et al., 2020). To make the models comparable, the same method was conducted to calculate the risk score of each OS sample in the TARGET cohort based on the corresponding genes in the 4 models. The Z-score method was applied to the preprocessing of risk scores, and samples with a risk score >0 were put into the high-risk group, while those with a risk score <0 were put into the low-risk group. The ROC and KM curves of the 3 comparison models are shown in Figure 12. The AUCs of these 3 models were lower than that of our 4-gene risk score model at 2, 3, and 5 years. Our 4-gene risk score model included a reasonable number of genes and had better performance. In addition, the 3 comparison models could significantly divide samples into high- and low-risk groups (*P* < 0.05).

### Epigenetic Modification of the 4 Genes

We analyzed the methylation of the 4 genes in the high- and low-risk groups, and the results showed that the level of methylation of *APBB1IP* in the high-risk group was higher than in the low-risk group, and the risk score heat map showed that in the high-risk group, *APBB1IP* was lowly expressed. The level of methylation of *FKBP11* in the low-risk group was higher than in the high-risk group, and the risk score heat map showed that *FKBP11* was lowly expressed in the low-risk group (Supplementary Figure 3).

Thus, the degree of methylation had a negative correlation with gene expression. The expression of *APBB1IP* and *FKBP11* was found to be regulated by methylation to a certain extent. There was no significant relationship between the expression of *GJA5* or *NPC2* and the level of methylation.

## Discussion

The treatment and prognosis of OS have not changed much in the past 30 years. Due to the poor prognosis of OS, it is necessary to determine prognostic biomarkers for OS patients in order to then use appropriate treatment strategies, including immunotherapy. We conducted a comprehensive study to establish a 4-gene signature, which was verified through internal and external testing cohorts. An immune-related risk model was established to explore the potential association between OS risk score and survival and to provide new biomarkers for OS prognosis.

In our study, the gene expression profiles and clinical data of 84 OS samples were obtained from the TARGET database, and 42 samples were randomly selected as the training cohort. Using the NMF algorithm to cluster molecular subtypes, two molecular subtypes (C1 and C2) were obtained, and 597 DEGs between C1 and C2 (including 552 upregulated genes and 45 downregulated genes) were identified. Functional and pathway enrichment analysis results showed that immune-related pathways, such as natural killer cell-mediated cytotoxicity, the toll-like receptor signaling pathway, the T cell receptor signaling pathway, the B cell receptor signaling pathway, and other immune-related pathways, were significantly enriched in the C1 subtype, suggesting that C1 was closely related to immunity. Moreover, the immune score of C1 was significantly higher than that of C2, and the prognosis of C1 was better than that of C2. Further, the results of the KEGG pathway analysis of the DEGs between the subtypes of the TARGET cohort showed that some immune-related pathways had a significant difference. Specifically, the HIF-1 signaling pathway was enriched. HIF-1α promotes tumor cell growth, migration, and invasion in OS through activation of the AKT/cyclin D1 signal cascade (Zhang et al., 2018). In recent years, immunotherapy has become a novel and effective treatment method, and it has been used in the treatment of various tumors, including OS (Lettieri et al., 2016; Wang et al., 2016; Thanindratarn et al., 2019). However, only a few patients receiving immunotherapy have responded to this treatment due to the immunosuppressive microenvironment (de Visser et al., 2006). Therefore, it is necessary to study the biomarkers that enable the prediction of the benefits of immunotherapy, which could aid clinical decision-making for individualized treatment.

A total of 14 genes associated with survival were obtained through univariate Cox analysis, and 8 genes were selected as target genes by LASSO regression. Further, the AIC was used to obtain 4 genes (*GJA5, APBB1IP, NPC2*, and *FKBP11*) to include in a 4-gene signature as a prognostic risk model. The results showed that high *FKBP11* expression was correlated with high risk (a risk factor), while high *GJA5, APBB1IP*, or *NPC2* expression was associated with low risk (protective factors).

FK506 binding protein 11 (FKBP11) belongs to the FK506 binding protein family (Rulten et al., 2006), and FKBP11 mRNA is present at high levels in many secreting tissues, including pancreas, stomach, and salivary gland tissues (Bonner and Boulianne, 2017). FKBP11 is involved in the regulation of mTOR (Hausch et al., 2013; Wang et al., 2019) and the pathogenesis of stress-related inflammatory diseases, including type 2 diabetes mellitus, systemic lupus erythematosus, and hepatitis (Ruer-Laventie et al., 2015; Wang et al., 2017, 2018). In addition, a gradual increase in *FKBP11* expression has been detected in the development of hepatocellular carcinoma (HCC), suggesting that FKBP11 may be a potential early biomarker for HCC (Lin et al., 2013). Gap junction alpha-5 protein (GJA5, also known as connexin 40) is a constitutive vascular gap junction protein that plays an important role in the coupling between vascular wall cells (Bai, 2014; Lu and Wang, 2017). New ultraviolet target genes, including *GJA5*, have been identified, and they are often dysregulated in human squamous cell carcinoma (Shen et al., 2017). However, the significance of GJA5 in tumorigenesis remains unclear. Amyloid beta precursor protein binding family B member 1 interacting protein [APBB1IP, also known as Rap1-GTP-interacting adaptor molecule (RIAM)], appears to play a role in signal transduction from Ras activation to actin cytoskeleton remodeling, and it seems to mediate Ras-related protein 1-induced adhesion (Bromberger et al., 2019). APBB1IP integrates signaling events that are critical for integrin-mediated immune function control and cancer progression (Patsoukis et al., 2019). Some studies have shown that APBB1IP is a new biological marker associated with gastric cancer and head and neck squamous cell carcinoma (Sanati et al., 2018; Shen et al., 2019). The Niemann–Pick type C2 (NPC2) protein is involved in the regulation of intracellular cholesterol homeostasis via direct binding with free cholesterol (Kamata et al., 2015). NPC2 is abundant in normal liver tissue, but it is downregulated in HCC. Further, low NPC2 levels may predict poor prognosis and regulate the progression of HCC by regulating the ERK1/2 pathway (Liao et al., 2015; Chen et al., 2018). To further understand the biological correlations of these genes, we conducted functional enrichment analysis and found that these genes were mainly concentrated in immune-related pathways. The 4-gene signature was reliable and performed well in predicting OS prognosis and the effect of immunotherapy.

Furthermore, the 4-gene signature was able to divide samples into high- and low-risk groups appropriately in terms of age, sex, and metastasis. The results suggested that the risk model had good predictive ability in terms of different clinical features and that the risk score could be used as an independent prognostic risk factor.

To observe the relationships between the risk scores of the different samples and biological functions, KEGG_JAK_STAT_SIGNALING_PATHWAY, KEGG_NATURAL_KILLER_CELL_MEDIATED_CYTOTOX ICITY, KEGG_TOLL_LIKE_RECEPTOR_SIGNALING_PATH WAY, and KEGG_B_CELL_RECEPTOR_SIGNALING_PATH WAY were selected. JAK/STAT signal transduction is an essential part of growth factor and cytokine signaling, which is involved in virtually all immune regulatory processes and has become an attractive target for many immune, inflammatory, and hematopoietic diseases (Waldmann and Chen, 2017; Gao et al., 2018; Owen et al., 2019). Natural killer cells are critical effector lymphocytes that mediate tumor immune surveillance and clearance and play an important role in innate and adaptive immune responses to tumors (Malmberg et al., 2017). Toll-like receptor signaling is involved in activating innate and adaptive immune responses, and there is substantial evidence of the benefit of targeting this pathway in cancer treatment (Li et al., 2014; Moradi-Marjaneh et al., 2018). B cell receptor signaling is critical for normal B cell development and adaptive immunity, and B cell receptor signal transduction supports the survival and growth of malignant B cells in patients with B cell leukemia or lymphoma (Burger and Wiestner, 2018). These pathways were all negatively correlated with risk score.

More studies of OS prognosis models are being reported as time goes on. We compared three published OG prognosis signatures to prove the superiority of our model. Goh et al. (2019) developed a novel OS prognostic score with grouped-variable selection using network-regularized high-dimensional Cox regression analysis. Zhang et al. (2019) built a predictive tool for OS lung metastasis and progression by using co-expression network analysis. Shi et al. (2020) constructed a 3-gene risk signature based on metastasis-associated genes for the prediction of OS prognosis and therapeutic targets. To make the models comparable, the same method was conducted to calculate the risk score of each OS sample in the TARGET cohort based on the corresponding genes in these 4 models. The AUCs of the 3 comparison models were lower than that of our 4-gene risk score model at 2, 3, and 5 years. Our 4-gene risk score model used a reasonable number of genes and had significantly high discriminatory power in predicting overall survival.

Our study has some limitations. Because the incidence of human OS is low, the number of OS samples in the TARGET database is relatively small. Only 84 OS samples were obtained, which may lead to selection bias. In addition, some important and meaningful genes may have been missed in our multiple screening processes. For better clinical applications, more samples are needed in future research to verify our findings. Therefore, it is necessary to do more functional research studies of the 4 genes mentioned in this paper. Furthermore, numerous clinical studies as well as animal and cell experiments should be carried out to verify the prediction accuracy of our risk model and discover potential immune-related mechanisms.

In conclusion, we constructed an effective 4-immune gene signature (including *GJA5, APBB1IP, NPC2*, and *FKBP11*) based on TARGET and GEO data for predicting OS prognosis, and the stability and accuracy of the model were assessed. Our work will help clinicians evaluate OS prognosis and select appropriate immunotherapy targets. In the future, this risk prognosis model should be further validated in more OS patients.

## Data Availability Statement

The original contributions presented in the study are included in the article/Supplementary Material, further inquiries can be directed to the corresponding author/s.

## Author Contributions

MC: conceptualization, methodology, software, and writing—reviewing and editing. JZ: data curation and writing—original draft preparation. HX: visualization and investigation. ZL, XC, and HW: software and validation. YS: supervision. All authors contributed to the article and approved the submitted version.

## Conflict of Interest

The authors declare that the research was conducted in the absence of any commercial or financial relationships that could be construed as a potential conflict of interest.

## Supplementary Material

Supplementary Figure 1 Immune-related pathways between C1 and C2.
