# Supplementary material for: Identification and Development of a Novel 4-Gene Immune-Related Signature to Predict Osteosarcoma Prognosis
Source: Front Mol Biosci. 2020 Dec 23;7:608368. doi: 10.3389/fmolb.2020.608368 (PMC7785859; doi:10.3389/fmolb.2020.608368)
Supplement: Supplementary file 1 [file Table_1.DOCX]

**Table 1. Sample Clinical Features**

| **Clinical Features** | **TARGET-OS** | **GSE21257** |
| --- | --- | --- |
| **Status** |  |  |
| Censored | 55 | 30 |
| Dead | 29 | 23 |
| **Gender** |  |  |
| Male | 47 | 34 |
| Female | 37 | 19 |
| **Metastatic** |  |  |
| YES | 21 | 34 |
| NO | 63 | 19 |
| **Age** |  |  |
| ≤ 15 | 46 | 21 |
| ＞15 | 38 | 32 |
