# Supplementary material for: Identification and Development of a Novel 4-Gene Immune-Related Signature to Predict Osteosarcoma Prognosis
Source: Front Mol Biosci. 2020 Dec 23;7:608368. doi: 10.3389/fmolb.2020.608368 (PMC7785859; doi:10.3389/fmolb.2020.608368)
Supplement: Supplementary file 2 [file Table_2.DOCX]

**Table 2: Univariable analysis and multivariable analysis of the entire TARGET**

| **Variables** | **Univariable analysis** | | | | **Multivariable analysis** | | | |
| --- | --- | --- | --- | --- | --- | --- | --- | --- |
|  | **HR** | **95% CI of HR** | | ***P*** | **HR** | **95% CI of HR** | | ***P*** |
|  |  | **lower** | **upper** |  |  | **lower** | **upper** |  |
| Age | 0.9901 | 0.9118 | 1.0750 | 0.8130 | 1.0191 | 0.9298 | 1.1170 | 0.6858 |
| Gender | 0.6870 | 0.3304 | 1.4290 | 0.3150 | 0.6521 | 0.3005 | 1.4150 | 0.2793 |
| Metastatic | 4.7400 | 2.2710 | 9.8950 | 3.4E-05 | 3.1864 | 1.4696 | 6.9090 | 0.0033 |
| RiskScore | 3.4030 | 2.1600 | 5.3610 | 1.3E-07 | 2.9103 | 1.8364 | 4.6120 | 5.4E-06 |
