# Supplementary figures and images for: Identification and Development of a Novel 4-Gene Immune-Related Signature to Predict Osteosarcoma Prognosis
Source: Front Mol Biosci. 2020 Dec 23;7:608368. doi: 10.3389/fmolb.2020.608368 (PMC7785859; doi:10.3389/fmolb.2020.608368)

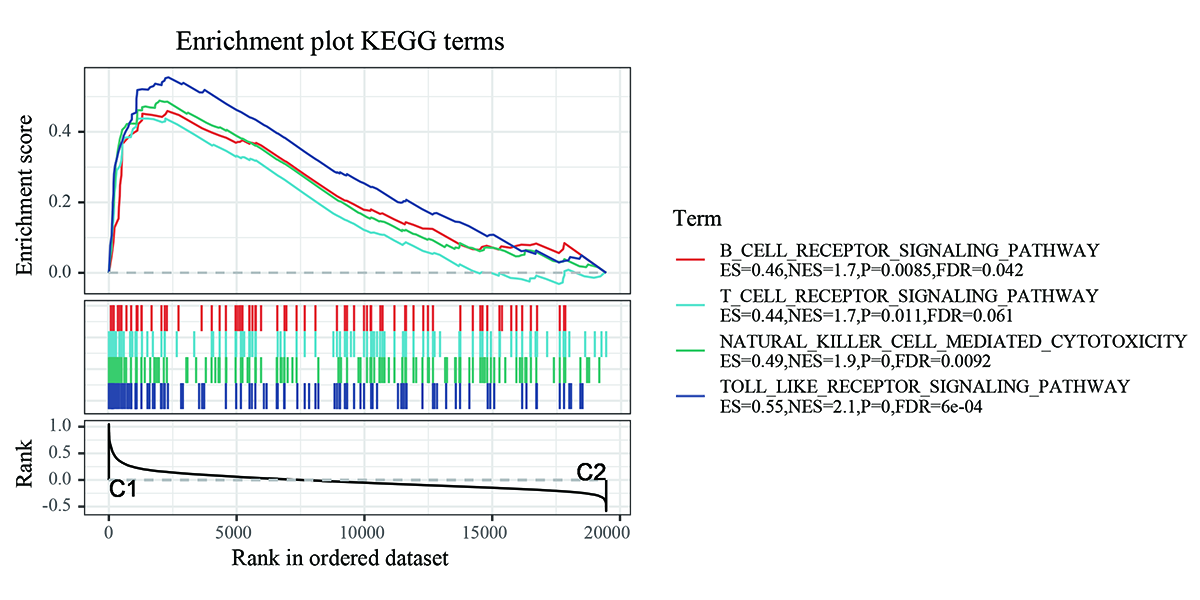

Supplement: Supplementary Figure 1 — Immune-related pathways between C1 and C2. [file Image_1.TIF]

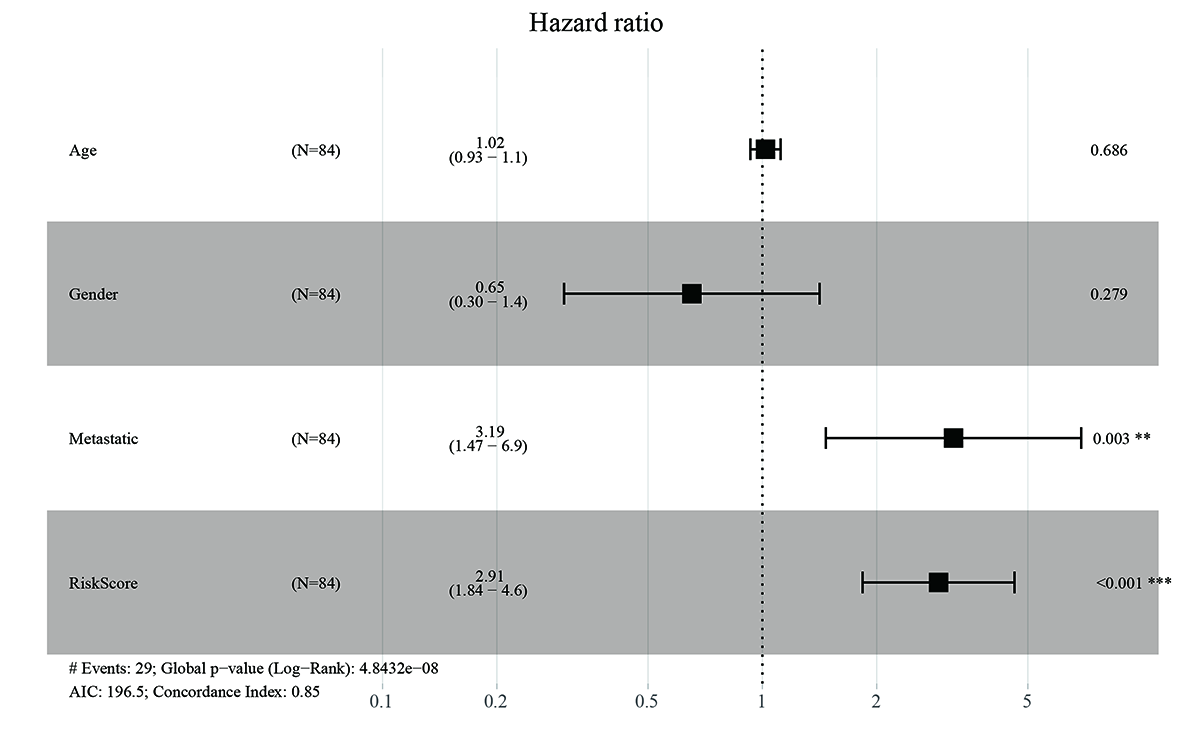

Supplement: Supplementary file 11 [file Image_2.TIF]

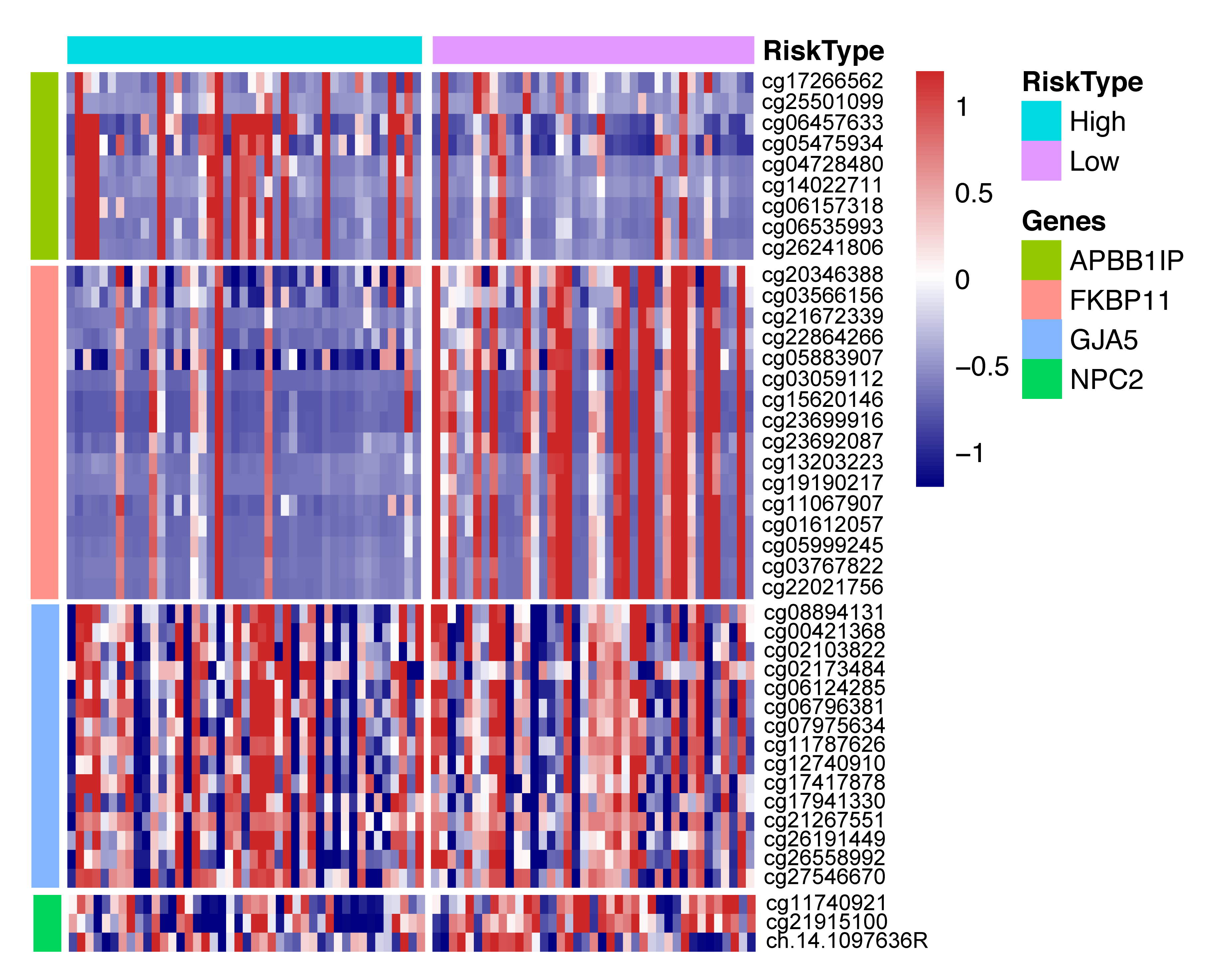

Supplement: Supplementary file 12 [file Image_3.TIF]
